# Characteristics of Soil Heavy Metal Pollution and Health Risk Assessment in Urban Parks at a Megacity of Central China

**DOI:** 10.3390/toxics11030257

**Published:** 2023-03-10

**Authors:** Ding Li, Qing Lu, Limei Cai, Laiguo Chen, Hanzhi Wang

**Affiliations:** 1 Hubei Key Laboratory of Petroleum Geochemistry and Environment, Yangtze University, Wuhan 430100, China; 2 Key Laboratory of Exploration Technologies for Oil and Gas Resources, Ministry of Education, Yangtze University, Wuhan 430100, China; 3 Guangdong Provincial Key Laboratory of Water and Air Pollution Control, South China Institute of Environmental Science, Ministry of Ecology and Environment, Guangzhou 510535, China; 4 School of Resources & Environmental Science, Wuhan University, Wuhan 430079, China

**Keywords:** urban park, pollution assessment, source apportionment, probabilistic health risk

## Abstract

In this study, we compared the concentrations of the heavy metals Cd, Cr, Cu, Zn, Ni, and Pb in the surface soils of urban parks in Wuhan, Hubei Province, with those in the surface soils of urban parks worldwide. The soil contamination data were assessed using enrichment factors and spatial analysis of heavy metals using inverse distance weighting and quantitative analysis of heavy metal sources with a positive definite matrix factor (PMF) receptor model. Further, a probabilistic health risk assessment of children and adults using Monte Carlo simulation was performed. The average Cd, Cr, Cu, Zn, Ni, and Pb concentrations in the surface soils of urban parks were 2.52, 58.74, 31.39, 186.28, 27.00, and 34.89 mg·kg^−1^, respectively, which exceeded the average soil background values in Hubei. From the inverse distance spatial interpolation map, heavy metal contamination was primarily observed to be present to the southwest of the main urban area. The PMF model resolved four sources: mixed traffic and industrial emission, natural, agricultural, and traffic sources, with relative contributions of 23.9%, 19.3%, 23.4%, and 33.4%, respectively. The Monte Carlo health risk evaluation model demonstrated negligible noncancer risks for both adult and child populations, whereas the health effects of Cd and Cr on children were a concern for cancer risks.

## 1. Introduction

The new urbanization and urban–rural integration of China have been developing rapidly. Concomitantly, a large quantity of heavy metals are being released into urban environments. Urban parks constitute a typical product of urbanization and are prime locations for urban residents to rest, exercise, and convene scientific, educational, and cultural gatherings. Pesticides and chemical fertilizers make up the primary source of heavy metals in the soil, which engenders the potential exposure of park users to these metals. The metals accumulating in urban soil can enter the body via ingestion, inhalation, and skin contact [1,2]. Children are more susceptible to the adverse impacts of metals owing to their poorer autoimmunity combined with frequent hand-to-mouth activities and a greater skin exposure and absorption rate than adults. The possible sources of heavy metal contamination in parks include transportation (vehicle emissions and wear and tear of parts), industry (coal combustion, smelting, and chemicals), waste incineration (domestic waste and electronic waste incineration), and weathering of buildings and infrastructure [3,4]. In addition, pesticides and chemical fertilizers can increase heavy metal contamination in soils [5]. Thus, determining the concentrations, characteristics, sources, and health risks of soil heavy metals in urban parks is vital for strategizing risk management in urban settings.

The excessive release of heavy metals and other pollutants into urban soils may engender ecological degradation and severe human health effects. For example, Zn and Cu can affect gastrointestinal function, cause kidney damage, and alter the balance of other elements in the body. Cd can severely damage the digestive system and lungs, with long-term exposure potentially causing bone fragility [6]. Ni exposure may increase the risk of developing lung, nose, throat, and prostate cancers [7,8]. Mild Cr exposure can cause itching and irritation of the nose and skin, and the inhalation of high doses can induce organ damage, hair loss, gastric ulcers, convulsions, and even death [9]. Pb enters the body through the respiratory and gastrointestinal systems and is transported throughout the body via blood circulation as protein complexes or ions. Long-term Pb exposure may cause increased blood pressure, anemia, miscarriage, and male reproductive disorders [8]. Based on the high toxicity levels of Pb, the regulatory limits of Pb as established by the US Environmental Protection Agency (EPA) are 15 ug/L and 0.15 ug/m^3^ for drinking water and air, respectively [10].

Health risk assessment (HRA) evaluates the potential of metals to cause adverse health effects. Monte Carlo simulations (MCS) assess the variability and uncertainty of data. Presently, MCS techniques are widely used to determine probabilistic risks. Several studies have reported that heavy metal contamination in park soils poses a high potential risk to human health. Furthermore, based on the cancer risk (CR) and noncancer risk (NCR) of heavy metals, Han et al. reported that heavy metals in urban park soils pose a higher risk for children than for adults [11]. Another risk assessment focused on megacity parks also showed elevated CR of heavy metals in soil [12]. Thus, heavy metals in soils in urban parks may engender serious health effects in park users.

This study aimed to (1) identify the current concentrations of six heavy metals (Cd, Cr, Cu, Zn, Ni, and Pb) in urban parks in Wuhan, Hubei Province, (2) analyze the pollution characteristics of soil heavy metals and compare them with those of soil heavy metals in different urban parks worldwide, (3) identify the possible heavy metal sources using Pearson correlation and positive definite matrix factor model (PMF) receptor model analyses, and (4) assess exposure risks using a probabilistic risk assessment model.

## 2. Materials and Methods

### 2.1. Study Area and Soil Sampling

Wuhan (113°41′–115°05′ E, 29°58′–31°22′ N) is located in central China, eastern Hubei Province, at the confluence of the Yangtze and Han rivers. Wuhan is a famous national historical and cultural city, substantial industrial, scientific, and educational base, and comprehensive transportation hub. The city comprises 13 administrative districts and covers an area of 8569.15 km^2^. The topography is concave from the north and south to the middle, making the topographic profile of the city basin shaped. The low-lying and flat central area has fertile soil along the river and lake. The typical subtropical monsoon climate experienced by this region is suitable for the growth and development of various crops.

**Figure 1:**
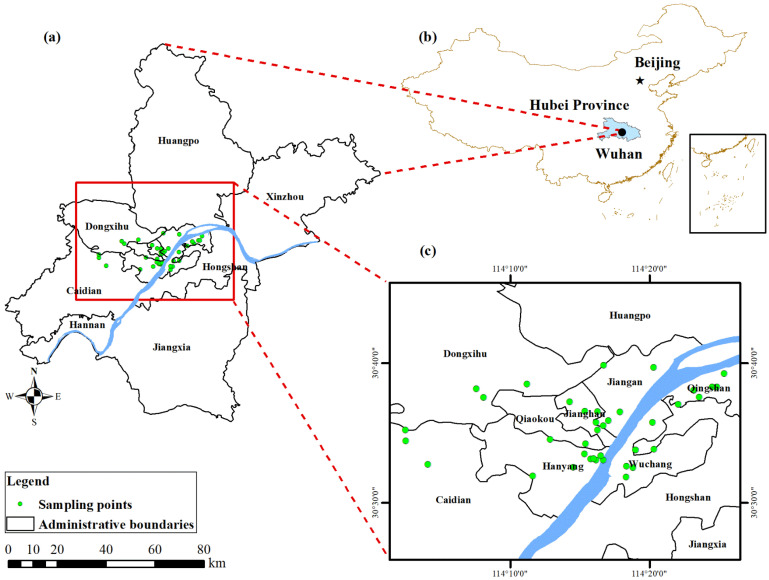
Geographical location of the soil sampling sites (**a**). China’s border, (**b**). Urban area, (**c**). Specific sampling point.

We identified 40 typical parks within Wuhan’s fourth ring road, an area with dense transport networks and a large population (Figure 1). The sampling sites were primarily selected from the green areas in the park where people usually spent more time and had more contact with the soil, such as the grassy areas near the park entrance where people entered and exited the park from and similar recreational areas where visitors usually gathered. We selected appropriate sampling methods for the park’s size. We collected 3–6 subsamples (0–5 cm) to mix thoroughly. We avoid local sources of pollution within 500 m, such as kilns, small chimneys, construction sites, etc. Root soil samples were collected and processed following the requirements of GB/T 36197-2018 issued by the Standardization Administration Committee. The soil samples were collected using a wooden shovel and placed in a polyethene plastic bag for sealed storage, which was labeled and sent to the laboratory for processing. The original sample mass was ≥1 kg. The sample mass after drying and sieving was >500 g.

### 2.2. Sample Analysis

The collected samples were dried, ground in an agate bowl, sieved to <0.15 mm, homogenized, and then solubilized using a concentrated acid mixture of HNO_3_-HF-HClO_4_. The concentrations of the soil heavy metals were assessed using an inductively coupled plasma–atomic mass spectrometer (NexION 2000, PerkinElmer, Waltham, MA, USA). All experimental reagents were of superior purity, and the measurements were quality-controlled using standard, parallel, and blank samples. Elemental recoveries ranged from 82% to 114%, and the relative measurement errors were within ±5%. The method detection limits for Cd, Cr, Cu, Zn, Ni, and Pb were 0.09, 2, 0.6, 1, 1, and 2 mg·kg^−1^, respectively.

### 2.3. Data Analysis

#### 2.3.1. Enrichment Factors

The enrichment factor (EF) method is a crucial index for quantitatively assessing soil contamination [13]. The reference element should be in a chemically stable form and at relatively high levels. Mn is abundant and stable in the earth’s crust. Therefore, this study uses Mn as the reference element. Formula (1) was used to calculate the EFs:

(1) $$\mathrm{EF}=\frac{{\left(C_{i}/C_{r}\right)}_{\mathrm{sample}}}{{\left(C_{i}/C_{r}\right)}_{\mathrm{background}}}$$

where *C_i_* is the concentrations of heavy metals in the soil and *C_r_* is the concentration of Mn (mg·kg^−1^). The method proposed by Sutherland was used to classify the contaminating heavy metals into different classes [14]. The correspondence between EF values and classification rank is shown in Table S1.

#### 2.3.2. Source Apportionment Model

PMF is a typical factor analysis receptor model widely used for analyzing contamination sources in soil, water, and air [15,16,17]. The calculation formulas of the model include:

(2) $$x_{ij}^{}=\sum_{k=1}^{p}g_{ik}f_{kj}+e_{ij}$$

(3) $$Q={\sum_{i=1}^{n}\sum_{j=1}^{m}\left(\frac{e_{ij}}{u_{ij}}\right)}^{2}$$

(4) $$c\;>\;\mathrm{MDL},\;u_{ij}=\sqrt{{\left(error\;fraction\times c\right)}^{2}+MDL^{2}}$$

(5) $$c\;\leq \;\mathrm{MDL},\;u_{ij}=5/6\times MDL$$

where *i*, *j*, and *k* indicate the numbers of samples, elements, and sources, respectively. *x_ij_* is the concentration of element *j* in sample *i* (mg·kg^−1^), *g_ik_* is the contribution of source *k* in sample *i* (mg·kg^−1^), *f_kj_* is the amount of element *j* from source *k*, *e_ij_* is the residual, *u_ij_* is the uncertainty of element *j* in sample *i*, MDL is the metal-specific method detection limit, and error fraction is the measurement uncertainty (%).

#### 2.3.3. Human Health Risk Assessment

The human health risk model for soil-heavy metals recommended by the US EPA is based on exposure via three routes⸺ingestion, inhalation, and skin contact [18,19]. We assessed the heavy metal exposure risk in two populations, children and adults. It is generally believed that hazard indices (HI) > 1 marks the presence of noncarcinogenic health risks, while HI < 1 represents no adverse health risks [20,21]. The risk threshold for carcinogenic risk (CR) is 10^−6^. A CR of <10^−6^ signifies negligible risk, CR between 10^−6^ and 10^−4^ denotes acceptable risk, and CR of >10^−4^ indicates unacceptable risk [22,23]. The HI and CR can be calculated using the following formulas:

(6) $$\mathrm{HI}=\sum_{}^{}\mathrm{HQ}=\sum \frac{{\mathrm{ADD}}_{ij}}{{\mathrm{RfD}}_{\mathrm{ij}}}$$

(7) $$\mathrm{TCR}=\sum_{}^{}\mathrm{CR}=\sum ADD_{ij}\times {\mathrm{SF}}_{\mathrm{ij}}$$

(8) $${\mathrm{ADD}}_{\mathrm{ing}}=C\times \frac{\mathrm{IngR}\times \mathrm{EF}\times \mathrm{ED}}{\mathrm{BW}\times \mathrm{AT}}\times 10^{-6}$$

(9) $${\mathrm{ADD}}_{\mathrm{dermal}}=C\times \frac{\mathrm{SA}\times \mathrm{AF}\times \mathrm{ABS}\times \mathrm{EF}\times \mathrm{ED}}{\mathrm{BW}\times \mathrm{AT}}\times 10^{-6}$$

(10) $${\mathrm{ADD}}_{\mathrm{inh}}=C\times \frac{\mathrm{InhR}\times \mathrm{EF}\times \mathrm{ED}}{\mathrm{PEF}\times \mathrm{BW}\times \mathrm{AT}}\times 10^{-6}$$

where HI and TCR are the hazard index (non-carcinogenic) and carcinogenic hazards, respectively; *RfD* is the toxicity threshold value (mg·kg^−1^·d), *SF* value (mg·kg^−1^·d) indicates the slope factor of heavy metals; ADD_ing_, ADD_dermal_, and ADD_inh_ are the average daily heavy metal exposure of ingestion, dermal absorption, and inhalation, respectively (mg·kg^−1^·d); C is the heavy metal concentration of the soil sample (mg kg^−1^); *RfD*, *SF*, and other related parameters are shown in Tables S2 and S3.

#### 2.3.4. Probabilistic Health Risk Assessment

Uncertain parameters in human health risk assessment models, including variation in environmental factors and differences in exposure patterns, may lead to the overestimation or underestimation of human health risks. Monte Carlo simulation is an iterative analysis process involving numerical calculations that uses random sampling and repeated calculations based on probability distribution and parameter ranges to minimize uncertainty and maximize reliability, thereby facilitating the interpretation of the risk assessment results. This technique was iterated 10,000 times to produce relevance, and the related parameters are shown in Tables S4 and S5.

## 3. Results and Discussion

### 3.1. Characteristics of Heavy Metal Concentrations

The statistical analysis of the six heavy metals⸺Cd, Cr, Cu, Zn, Ni, and Pb⸺in the park soil samples from Wuhan showed concentration ranges of 0.08–17.32, 22.61–109.54, 10.50–60.23, 92.60–398.83, 9.52–59.27, and 15.51–66.08 mg·kg^−1^, respectively (Table 1). The coefficients of variation of the heavy metals ranged from 34% to 153%, in descending order of Cd > Pb > Ni > Cu > Zn > Cr. The coefficient of variation of Cd was 153%, indicating substantial disturbance of Cd by human activities. The coefficients of variation of the remaining five heavy metals were in the range of 30–40%, indicating that the concentrations of Cr, Cu, Zn, Ni, and Pb in the soil were relatively uniformly distributed and less affected by human activities. Compared with the local soil background values and related control standards, the average concentrations of the heavy metals Cr and Ni were lower than those in Hubei Province [24]. In comparison, the average concentrations of Cd, Cu, Zn, and Pb were higher than those in the Hubei Province. According to the GB15618-2018 soil environmental quality risk management standard for agricultural land [25], the average concentrations of Cr, Cu, Zn, Ni, and Pb in the soil samples collected from the study areas were less than the risk screening values of agricultural land, the average value of Cd concentration was higher than the screening value of soil pollution risk, and the Cd concentration was >4 mg·kg^−1^ in eight samples; the results showed that Cd accumulation was the highest, suggesting severe contamination from an agricultural perspective. Hence, the heavy metal pollution in the soil of urban parks is noteworthy, especially for Cd, followed by Cu, Zn, and Pb.

**Figure 2:**
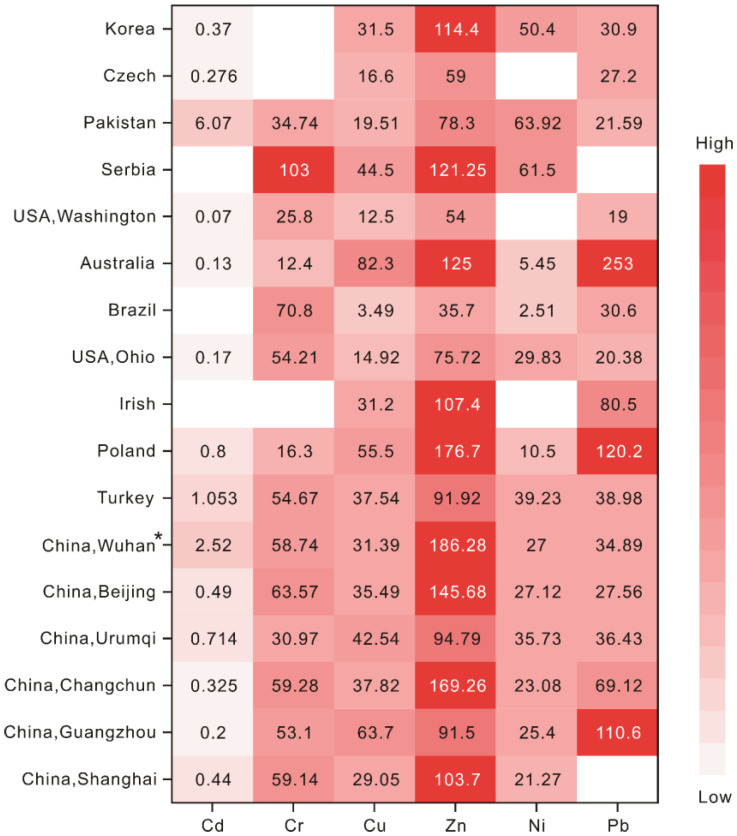
Comparative analysis of soil heavy metals in Wuhan*(this study) and in different regions (mg·kg^−1^).

The soil concentration of the study area and the urban parks of different countries in the world are shown in Figure 2 (Table S6 for details), and the limited data show that the existing heavy metal content data of urban parks at home and abroad are compared. The Cd and Zn in the soil of Wuhan Park, China, are significantly higher than those in Brazil, the Czech Republic, Washington, and other places [26,27,28]. The content of Cu, Cr, Ni, and Pb elements is at a low level compared with Australia, Serbia, Pakistan, and other places [3,29,30], which may be affected by each country’s industrial development level or geological background. It can also be seen that the content levels of metal Cr and Ni in Wuhan urban parks are generally equivalent to the content levels of other cities in China [31,32], indicating that the distribution of these two soil elements in various regions of China is relatively uniform, which shows that Cr and Ni elements are affected mainly by soil forming factors, geological conditions, and natural factors.

### 3.2. Heavy Metal Enrichment Factors

**Figure 3:**
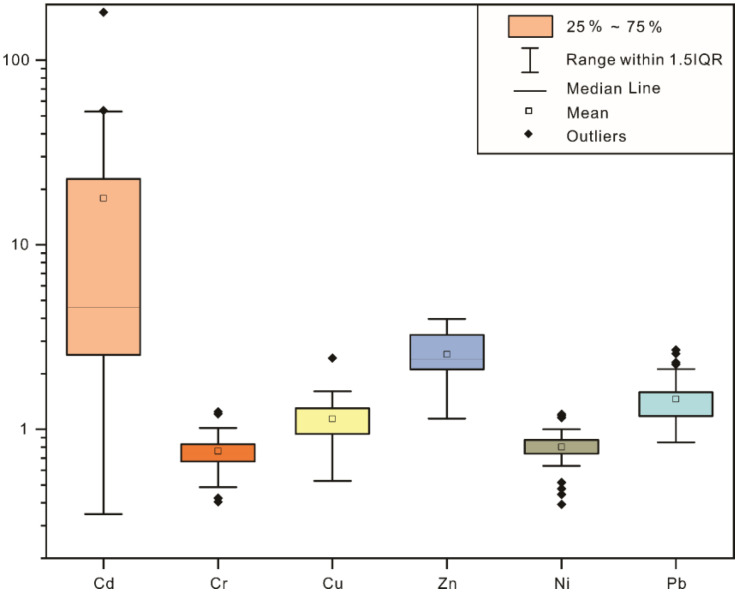
Box diagram of the distribution of metal enrichment factors in soil.

EF was calculated to characterize the role of anthropogenic activities on the concentration levels of heavy metals in urban park soils. As shown in Figure 3, the EFs of Cr and Ni in the sampling sites were between 0.6 and 1.2, indicating that these metals were mainly influenced by natural factors, such as soil-forming parent material and natural weathering. Conversely, the EFs of Cu and Zn were mainly distributed between 1.2 and 1.8, with some estimates exceeding 1.8, suggesting the influences of exogenous factors. Most EFs of Pb were distributed between 2.0 and 4.0. The EF for a small number of samples was >4. Notably, 90% of sample sites exhibited an EF of >2, 52.5% exhibited an EF of >5, and a few sites displayed an EF of >10. The study area was thus considerably disturbed by anthropogenic activities.

### 3.3. Spatial Distributions of Heavy Metals

**Figure 4:**
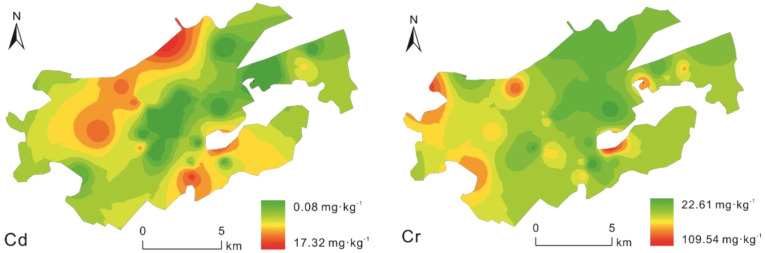

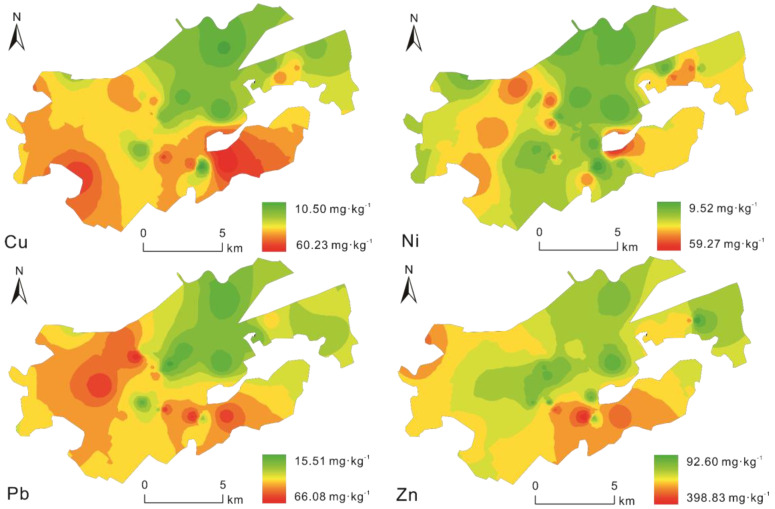
Spatial distribution of soil heavy metals in the study area.

The spatial distribution map of the concentration of each metal was created per the inverse distance interpolation method of heavy metals in the soil sample sites of the parks in the main urban areas of Wuhan, and the spatial distribution of the heavy metals in the main urban area was analyzed (Figure 4). The spatial distribution of Cr and Ni in the study area was relatively uniform, and the high-concentration areas were randomly distributed. The pollution sources of these metals were likely nonpoint, such as climate and parent soil materials.

The study areas with high Cd concentrations were approximately distributed in the south and north of the central city. The northern part encompasses the Gutian Industrial and Tijiao Industrial Parks. Gutian Industrial Zone is an important industrial town with a long history, mainly for light, chemical, and small- and medium-sized machinery. Tijiao Park is a chemical industrial area with chemical plants and raw material factories. The industrial activities in these areas considerably affect the soil concentrations of Cd [33,34].

Intensive traffic flow is the primary source of contamination in the southern area. Cu, Zn, Pb, and Cd are widely distributed in the southeast Wuchang District. Wuhan City is the largest city in central China, which, accompanied by the Wuhan landmark building, Huanghelou, a scenic attraction, influences its high motor vehicle ownership. The area experiences high traffic volume, automotive paint corrosion, corrosion-resistance material weathering, and brake wear, which potentially release heavy metals into the surrounding environment [35,36,37]. The results show that the wearing of automobile brake pads is the source of the largest heavy metal pollution load of Cu, followed by Zn > Pb > Cr > Cd. The largest pollution load of Zn is from the wear of automobile tires, followed by Pb, Cr, Cu, Ni, and Cd [38].

High Pb concentrations were detected in the vicinity of Gutian’s industrial area, which can be attributed to the atmospheric deposition of industrial emissions [39]. Therefore, no significant pollution sources were identified in the central part of the study area; primary metal pollution sources were located in the northern and southern parts of the chief urban area of Wuhan. Heavy metals in these latter areas were primarily influenced by industrial and traffic sources.

### 3.4. Source Apportionment of the Heavy Metals

#### 3.4.1. Correlation Analysis

As shown in Table 2, a significant positive correlation was observed between Cr–Ni, Cr–Cu, and Cr–Pb (r > 0.50), indicating that the sources of these heavy metals may be same or similar. The correlation coefficients of Cr–Ni and Cr–Cu were greater (r > 0.75) than of those mentioned above, thereby suggesting high homology. The correlations between Cd and other metals were not apparent, indicating that Cd sources differed from those of the other metals. The extremely high coefficient of variation of Cd also confirmed this conjecture. The correlation coefficients for Cu–Zn, Cu–Ni, and Cu–Pb were golden ratios, 0.618, 0.651, and 0.702, respectively, which passed the significance test at 0.01. The correlation coefficient for Zn–Pb was 0.533, indicating probable Zn and Pb homology.

#### 3.4.2. Analysis of PMF Model Sources

**Figure 5:**
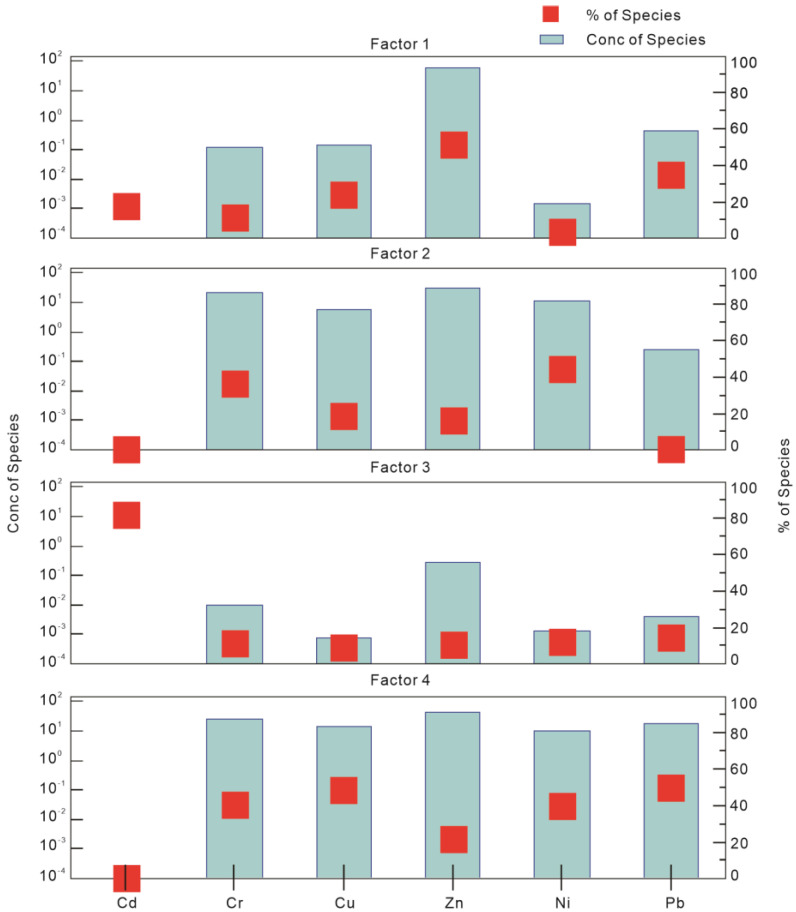
Pollution source diagram of heavy metal PMF analysis in study area.

A PMF model was used to identify and distribute sources of heavy metals in soil [40,41]. We used EPA PMF 5.0 software, employing set factor (2–6) and operation (20) numbers to compare the Q_r_/Q_e_ values one by one for different factors to determine the optimal factor number. Q_r_ is the optimal solution for the objective function Q obtained via PMF in the robust mode, and Q_e_ is the actual value of the objective function. The best model was obtained with four as the number of words using the actual and predicted content values. (Figure S1). The fitting coefficients of Cr and Cu exceeded 0.8, and those of Cd, Ni, Pb, and Zn exceeded 0.90. The PMF model resolved four factors corresponding to the diverse sources of heavy metals. The contribution of heavy metal factors is shown in Figure S2, as is observed in the source contribution diagram (Figure 5).

Factor 1 mainly contributes Zn (51.7%) and Pb (34.9%) and the metals were moderately enriched. Numerous studies have reported that traffic emissions constitute a critical source for the accumulation of these metals in soil [42,43,44]. In addition, contaminants produced owing to activities such as coal burning, mining, and metal smelting contribute to the accumulation of Pb and Zn in soil [45]. Wuhan Iron and Steel Company is the core company in the Qingshan District, Wuhan. Metallurgical companies, thermal power plants, cement plants, petrochemical plants, and other enterprises also operate in this area. Thus, Factor 1 constitutes a mixed source of transportation and industrial activity emissions.

Cr (36.7%) and Ni (46.7%) have the highest contribution rate in Factor 2, yet the average levels of these heavy metals were lower than the soil background values. Moreover, the coefficients of variation did not exceed 0.5, suggesting little impact of human activities on the concentrations of Cr and Ni. Liu et al. and Lv et al. also noted that the concentrations of Cr and Ni were primarily controlled by natural factors, such as parent soil material and soil-forming process [46,47]. Thus, Factor 2 is defined as a natural source.

The Factor 3 element was Cd, accounting for 82.4% of weight, which was found to exhibit a considerable pollution load in the study area. The average concentration of Cd was 14.82 times the soil background value. External Cd sources include the uncontrolled application of chemical fertilizers and pesticides [48]. Cd concentrations are relatively high, especially in calcium superphosphate fertilizer [49]. For enhanced decorative effect of vegetation in urban parks, a large amount of chemical fertilizer is required at the beginning of plantation. Vegetation is also often sprayed with pesticides in the later stages of growth to prevent diseases and pests. The supervision of chemical fertilizer and pesticide applications is weak during the early stage of development in China, thereby allowing Cd to directly enter the soil. Hence, severe Cd pollution may limit the use of land for agriculture.

In Factor 4, Pb, Cu, Cr, and Ni constituted the chief metals, contributing 50.1%, 48.1%, 40.5%, and 39.6% of weight, respectively. Many studies have shown that automobile exhaust is a quintessential source of Pb in urban soil, attributable to the addition of antiknock agents to gasoline [50,51,52,53]. Cu is widely used in vehicle braking systems and heat sinks owing to its high corrosion resistance and thermal conductivity [54]. Cr, Ni, V, Ti, and other elements are often added to motor vehicle alloys to improve performance. Thus, Cu, Cr, and Ni are released from the corrosion and wear and tear of motor vehicle parts to a certain extent [55,56]. Therefore, Factor 4 primarily constitutes traffic sources.

**Figure 6:**
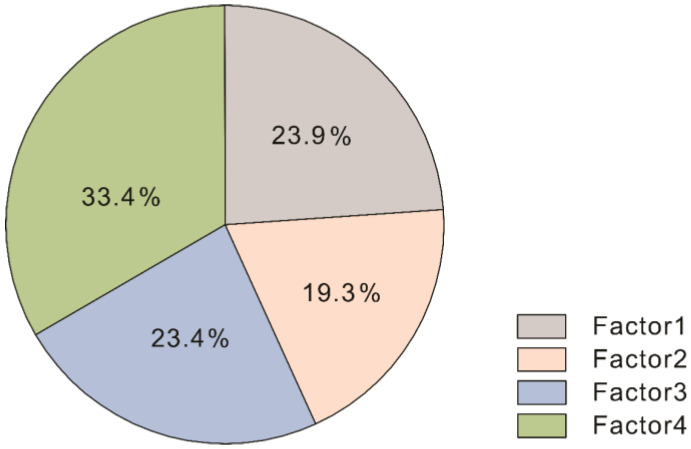
Proportion of heavy metal pollution sources.

According to the above discussion, there were four sources of heavy metal contamination in the study area, namely, diverse traffic and industrial emission, natural, agricultural, and traffic sources, contributing 23.9%, 19.3%, 23.4%, and 33.4%, respectively (Figure 6). Cd constitutes the main pollutant. The PMF source analysis model was used to determine the important sources of heavy metals in the soil, which lacked direct observational evidence. Subsequent studies could add other analytical indicators, such as isotopes and other geochemical means, to attain a more comprehensive identification of heavy metal sources.

### 3.5. Probabilistic Health Risks Assessment

#### 3.5.1. Noncancer Risk Assessment

**Figure 7:**
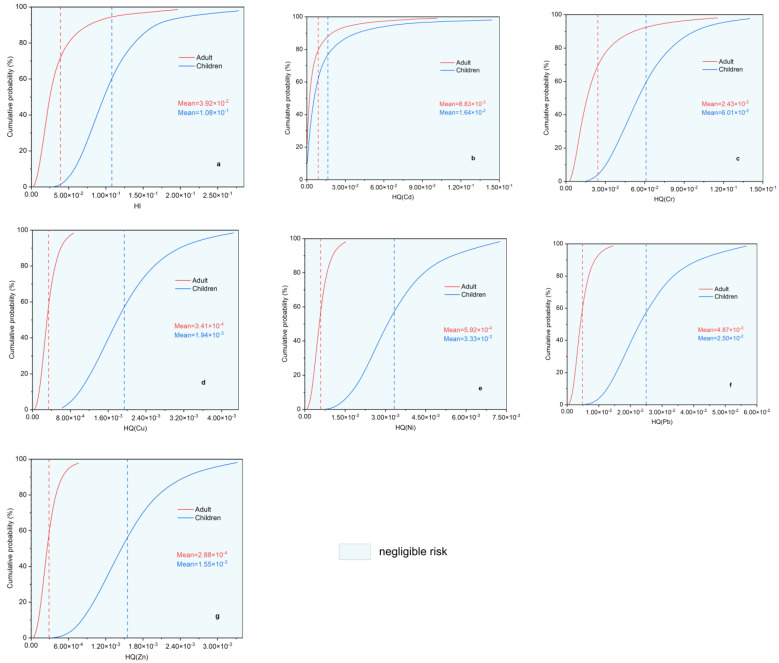
Probability distribution for (**a**) hazard index (HI) and hazard quotient (HQ) of (**b**) Cd, (**c**) Cr, (**d**) Cu, (**e**) Ni, (**f**) Pb, and (**g**) Zn. The red and blue vertical dashed lines represent mean values for adults and children, respectively.

NCR and CR due to exposures to heavy metals in park soil via three routes were estimated using Monte Carlo simulation (Table S7). No significant NCR was identified for any heavy metal for all the populations. The probability distributions of HI and hazard quotients (HQ) (Figure 7a–g) showed a mean HI for all populations of less than the guideline value of 1 in EPA. Thus, the risk of noncancer health impacts was minimal for the heavy metals. Children experience higher cumulative NCR than adults [57,58] owing to the frequent hand-and-foot activity and unique behavioral and physiological characteristics, such as parorexia, finger sucking, and high respiratory rate, exhibited by children. For adults and children, the trends in HQs are Cr > Cd > Pb > Ni > Cu > Zn and Cr > Pb > Cd > Ni > Cu > Zn, respectively. On comparing the two populations, the NCRs of Cr, Cd, and Pb were found to be significantly greater than those of Ni, Cu, and Zn.

#### 3.5.2. Cancer Risk

**Figure 8:**
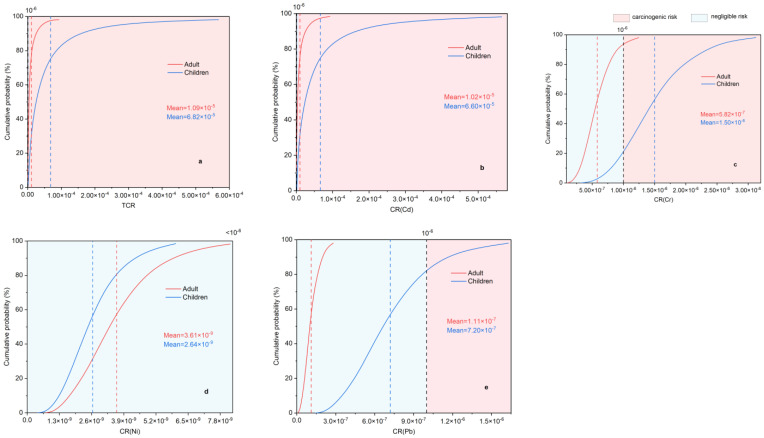
Probability distribution of (**a**) total cancer risk (TCR) and CR indices of (**b**) Cd, (**c**) Cr, (**d**) Ni, and (**e**) Pb. The red, blue, and green vertical dashed lines represent the mean values and the black line represents the acceptable threshold (1 × 10^−6^).

According to the probability distributions shown in Figure 8a–e, the total CR (TCR) of the four heavy metals (Cd, Cr, Ni, Pb) studied herein could not be considered risk-free, and each metal exhibited a different CR value. The mean TCR of adults and children were 1.09 × 10^−5^ and 6.82 × 10^−5^, respectively, (Figure 8a–e), which exceeded the acceptable threshold of 1 × 10^−6^. The 95% confidence interval for childhood TCR was 2.92 × 10^−6^ to 4.35 × 10^−4^ (Figure 8a and Table S5), which is roughly 68 times the acceptable threshold of 1 × 10^−6^. Moreover, 100% of TCR values for the child and adult populations exceeded 1 × 10^−6^ (Figure 8a and Table S7), indicating a non-negligible CR. Furthermore, on comparing the maximum mean CR values of the metals, Cd and Cr were identified as the major metals responsible for CR. In both populations, the mean CR of Cd and Cr exceeded the acceptable threshold of 1 × 10^−6^. The CR of Cd for children was 2.72 × 10^−6^ at the 10th percentile, which exceeded the risk threshold. Furthermore, based on the percentage of risk that exceeded the 1×10^−6^ threshold, Cd was associated with the highest CR in all populations.

Cd may also seriously damage the digestive system and lungs, with prolonged exposure potentially causing kidney disease, bone fragility, and other diseases [10]. Therefore, risk assessments for urban parks in China should address the exposure risk of heavy metals to children, especially those of Cd and Cr.

#### 3.5.3. Impacts and Recommendations

**Figure 9:**
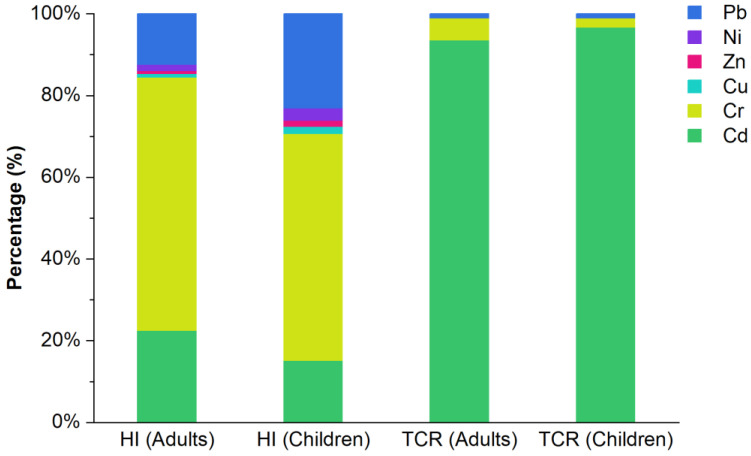
Heavy metal contribution for hazard index and cancer risk.

The contribution ratios of the six heavy metals for HI and CR are shown in Figure 9. The mean contribution ratios for HI indicated that Cd, Cr, and Pb significantly contributed to the NCR in the region, accounting for 96.9% and 94.0% in adults and children, respectively. Similarly, the contributions for Cd and Cr reached 98.9% and 99.0% in adults and children, respectively. In addition, among the six metals, Cr and Cd exhibited the highest NCR and CR, respectively. Overall, the mean HQ values for both adults and children were <1, indicating no significant adverse health hazard. Similarly, the mean CR results suggested no unacceptable cancer health hazards for Cr, Ni, and Pb in adults and Ni and Pb in children. Cd exposure in adults and Cr and Cd exposure in children were at acceptable risk levels. For all populations, the human health risks of the six heavy metals in the study area were within the acceptable range of 1 × 10^−6^ to 1 × 10^−4^, suggesting no significant health hazard.

Urban parks are essentially created for the rest, exercise, and recreation of urban residents and for holding science and education conventions. Hence, characterizing the distribution pattern of heavy metals in the soil of urban parks, determining their sources, and assessing their health risks will help implement relevant pollution remediation measures as it has an essential impact on the health of urban residents [59]. The soils in some areas of urban parks exhibit heavy metal contamination, posing a certain degree of health risk. The concentrations of these heavy metals are primarily affected by industrial emissions, traffic emissions, and uncontrolled use of fertilizers and herbicides. Thus, the scientific management of urban parks by the Landscape Bureau should be strengthened. Further, decision-makers should eliminate the production and utilization of substandard fertilizers and herbicides to reduce the entry of heavy metals into soils at the source. Tall and dense ornamental trees can be planted around urban parks to reduce the direct input of metals from traffic emissions.

Measures should also be taken to strengthen the compliance of industrial enterprises with smoke and dust emission standards. Industrial activities that require burning fossil fuels should employ alternative energy sources as much as possible. For protecting human health, necessary remediation measures need to be taken for the soil areas exhibiting the most severe heavy metal contamination [60]. Phytoremediation is a standard tool for soil remediation. Lime and soil conditioners (e.g., diatomaceous earth) can also increase soil pH and raise organic matter concentrations [61,62], thereby reducing bioavailability of heavy metals in the soil.

## 4. Conclusions

Herein, we determined the characteristics, sources, and health risks of heavy metals in urban parks in a populated central Chinese megacity. Cd, Cu, Zn, and Pb contamination was observed to varying degrees. Cd was associated with the most severe contamination, exhibiting higher health risks than the other heavy metals. The heavy metal concentrations of this study were higher than or similar to the concentrations in other parks worldwide. The spatial distribution of heavy metals supported PMF source analysis. Monte Carlo simulation was used for probabilistic HRA, and the HQs for children and adults were determined to be below the acceptable thresholds. Cd made the greatest contribution to TCR.

PMF source analysis enables the source identification of heavy metals and allows the quantitative analysis of channel contribution in addition to probabilistic risk assessment using Monte Carlo simulation, which minimizes uncertainty factors for the accurate identification of high-risk heavy metals. However, we conducted HRA based on the available concentration data, ignoring the influences of other factors, such as soil type, particle size, and pH, on heavy metal concentrations and bioavailability. Hence, the relationships between heavy metal contamination status and soil physicochemical properties must be investigated to reduce risk assessment errors.

## Figures and Tables

**Table 1:** Statistical summary of soil heavy metal concentrations (mg·kg^−1^) in urban parks in Wuhan.

Element	mg·kg^−1^						CV%	ER%
	MIX	MAX	Mean	SD	ABV	Guide Value		
Cd	0.08	17.32	2.52	4.31	0.17	0.60	153	90
Cr	22.61	109.54	58.74	20.18	86	250	34.4	5
Cu	10.50	60.23	31.39	11.77	30.7	100	37.5	50
Zn	92.60	398.83	186.28	66.92	83.6	300	35.9	100
Ni	9.52	59.27	27.00	10.49	37.3	190	38.9	10
Pb	15.51	66.08	34.89	13.60	26.7	170	39.0	67.5

Abbreviations: SD, standard deviation; ABV, average background value; CV, coefficient of variation; ER, exceedance rate, and the proportion of metal elements exceeding ABV at sample sites Guide values were according to the GB15618-2018 soil environmental quality: risk control standard for soil contamination of agricultural land. It recommended screening values that were the standard values of soil contamination. Exceeding the standard may pose a risk to human health.

**Table 2:** Pearson correlation coefficients for soil heavy metals.

	Cd	Cr	Cu	Zn	Ni	Pb
Cd	1					
Cr	0.155	1				
Cu	0.058	0.755 **	1			
Zn	−0.05	0.484 **	0.618 **	1		
Ni	0.243	0.840 **	0.651 **	0.286	1	
Pb	0.229	0.517 **	0.702 **	0.533 **	0.383 *	1

Note: * *p* < 0.05; ** *p* < 0.01.

## Data Availability

Data are contained within the article and Supplementary Materials.

## Supplementary Materials

The following supporting information can be downloaded at: https://www.mdpi.com/article/10.3390/toxics11030257/s1, Figure S1: Fitting coefficients (R2) between soil heavy metal(loid)s observed concentrations and predicted concentrations by PMF model; Figure S2: Factor contributions of soil heavy metal(loid)s from PMF model; Table S1: Correspondence between enrichment factor (EF) values and classification rank; Table S2: Corresponding reference dose (RfD) and slope factors (SF) values of metals in soil by different exposure pathways used in health risk assessment model with Mote Carlo simulator; Table S3: Values of each parameter in the human health risk assessment; Table S4: Uncertain concentrations (mg·kg−1) of heavy metal(loid)s in soils in parks; Table S5: The related parameters used in the Monte Carlo simulation; Table S6: Statistical summary of heavy metal(loid) concentrations (mg·kg−1) in surface soils of this study and global urban parks given in literature; Table S7: Summary statistics for non-carcinogenic and carcinogenic health risk range based on Monte Carlo simulation using Crystal Ball (vs. 11.1.2.4). References [63,64,65,66,67,68,69,70,71,72,73,74,75,76,77,78,79] are cited in the supplementary materials.
