# Supplementary material for: Characteristics of Soil Heavy Metal Pollution and Health Risk Assessment in Urban Parks at a Megacity of Central China
Source: Toxics. 2023 Mar 10;11(3):257. doi: 10.3390/toxics11030257 (PMC10053100; doi:10.3390/toxics11030257)
Supplement: Supplementary file 1 [file toxics-11-00257-s001.zip › toxics-2240534-supplementary.pdf]

Supplementary Materials

Characteristics of Soil Heavy Metal Pollution and Health Risk Assessment in Urban Parks at a Megacity of Central China

Ding Li [1], Qing Lu [1,3], Li-Mei Cai [1,2,*], Lai-Guo Chen [3,*] and Han-Zhi Wang [1,2]

[1] Hubei Key Laboratory of Petroleum Geochemistry and Environment, Yangtze University, Wuhan 430100, China
[2] Key Laboratory of Exploration Technologies for Oil and Gas Resources, Yangtze University, Ministry of Education Wuhan 430100, China
[3] Guangdong Provincial Key Laboratory of Water and Air Pollution Control, South China Institute of Environmental Science, Ministry of Ecology and Environment, Guangzhou 510535, China
* Correspondence: clmktz88@yangtzeu.edu.cn (L.-M.C.); chenlaiguo@scies.org (L.-G.C.)

Text S1 The details of average daily dose (*ADD*)

The average daily dose (*ADD*) of each metal was calculated as follows (Adedeji, 2020; Chai et al., 2021):

$$ADD_{ing} = \frac{C \times IngR \times EF \times ED}{BW \times AT} \times 10^{-6} \quad (4)$$

$$ADD_{der} = \frac{C \times SA \times AF \times ABS \times EF \times ED}{BW \times AT} \times 10^{-6} \quad (5)$$

$$ADD_{inh} = \frac{C \times InhR \times EF \times ED}{PEF \times BW \times AT} \quad (6)$$

Where ADD_{ing}、ADD_{der} and ADD_{inh} are the average daily heavy metal exposure of ingestion, dermal absorption, and inhalation, respectively (mg·kg^{-1}·d); *C* is the heavy metal concentration of soil sample (mg kg^{-1}). The values of each parameter used in the human health risk assessment are shown in Table S2 and S3.

Table S1 Correspondence between enrichment factor (EF) values and classification rank.

Contamination level	Value
**Enrichment factor**	
No enrichment	EF≤1
Minimal enrichment	1 ＜ EF ≤ 2
Moderate enrichment	2 ＜ EF ≤ 5
Significant enrichment	5 ＜ EF ≤ 20
Very high enrichment	20 ＜ EF ≤ 40
Extremely high enrichment	EF ≥ 40

Table S2 Corresponding reference dose (*RfD*) and slope factors (*SF*) values of metals in soil by different exposure pathways used in health risk assessment model with Mote Carlo simulator.

elements	*RfD*（mg/(kg · d)）			*SF*（(kg · d)/mg）		
	ingestion	inhalation	dermal contact	ingestion	inhalation	dermal contact
Cu	4.00E-02[a]	4.02E-02[a]	1.20E-02[a]	n/a	n/a	n/a
Cr	3.00E-03[a]	2.86E-05[a]	6.00E-05[a]	8.50E-03[b]	4.20E+01[a]	n/a
Ni	2.00E-02[a]	2.06E-02[a]	5.40E-03[a]	n/a	8.4E-01[a]	n/a
Zn	3.00E-01[a]	3.00E-01[a]	6.00E-02[a]	n/a	n/a	n/a
Pb	3.50E-03[a]	3.52E-03[a]	5.25E-04[a]	8.50E-03[c]	n/a	n/a
Cd	1.00E-03[a]	1.00E-05[a]	1.00E-05[a]	6.1E+00[b]	6.30E+00[a]	n/a

n/a, data not available. [a] Gu et al. (2017), [b] Yang et al. (2019), [c] USEPA, (2019), [c] Ferreira-Baptista and De Miguel (2005)

Table S3 Values of each parameter in the human health risk assessment.

Parameter	Definition	Unit	Adults	Children	Reference
IngR	Ingestion rate	$mg \cdot day^{-1}$	20	50	USEPA, 2011
InhR	Inhalation rate	$m^3 \cdot day^{-1}$	16	7.6	USEPA, 2011
EF	Exposure frequency	$day \cdot a^{-1}$	350	350	USDoE, 2011
ED	Exposure duration	a	24	6	USEPA, 2011
BW	Average body weight	kg	56.8	15.9	Zhang, 2021
AT	Average time	day	8760	2190	USEPA, 2011
PEF	Particle emission factor	$m^3 \cdot kg^{-1}$	1.36E+09	1.36E+09	USEPA, 2011
SA	Exposured skin area	cm^2	5700	2800	USEPA, 2011
AF	Skin adherence factor	$kg \cdot cm^{-2} \cdot day^{-1}$	2.00E-07	1.00E-06	USEPA, 2011
ABS	Dermal absorption factor	no unit	0.001	0.001	USEPA, 2011

Table S4 Uncertain concentrations (mg·kg^{-1}) of heavy metal(loid)s in soils in parks.

Heavy metals	Probabilistic distribution	Parameters LN (mean, SD)	Reference
Cu	Lognormal	LN (31.39, 10.80)	This study
Cr	Lognormal	LN (58.74, 18.42)	This study
Ni	Lognormal	LN (27.00, 9.17)	This study
Zn	Lognormal	LN (186.28, 58.72)	This study
Pb	Lognormal	LN (34.89, 12.84)	This study
Cd	Lognormal	LN (4.46, 13.74)	This study

(Here, the software Crystal Ball (11.1.2.4, Oracle, USA) was used to fit the probabilistic distribution of the uncertain concentrations of heavy metal(loid)s.)

Table S5 The related parameters used in the Monte Carlo simulation.

Parameter	Unit	Probabilistic distribution	Value	Reference
C	mg kg^{-1}	Log-normal	This study	-
BW	kg	Log-normal (children)	LN (37.0, 2.98)	MEP, 2013
		Uniform (adults)	UN (55.7, 68.6)	MEP, 2013
EF	day year^{-1}	Triangular	TRI (180, 345, 365)	Yang, 2022
SA	cm^2	Triangular (children)	TRI (430, 860, 2160)	Liu et al., 2021
		Triangular (adults)	TRI (760, 1530, 3820)	Liu et al., 2021
AF	mg cm^{-2} day^{-1}	Log-normal (children)	LN (0.49, 0.54)	Chen et al., 2022
		Log-normal (adults)	LN (0.65, 1.20)	Chen et al., 2022
IngR	mg day^{-1}	Triangular (children)	TRI (66, 103, 161)	USEPA, 2011
		Triangular (adults)	TRI (4, 30, 52)	USEPA, 2011
InhR	m^3 day^{-1}	Log-normal (children)	LN (7.19,1.62)	Chen et al., 2016
		Log-normal (adults)	LN (16.57,4.05)	Chen et al., 2016

Note: LN (mean, SD); UN (min, max); TRI (minimum, best, maximum)

Table S6 Statistical summary of heavy metal(loid) concentrations (mg·kg^{-1}) in surface soils of this study and global urban parks given in literature.

City	Year	Park number	Cd	Cr	Cu	Zn	Ni	Pb	Reference
Korean	2017	14	0.37	n/a	31.5	114.4	50.4	30.9	Kim et al. 2017
Czech	2019	1	0.276	n/a	16.6	59	n/a	27.2	Brtnick et al. 2019
Pakistan	2016	11	6.07	34.74	19.51	78.3	63.92	21.59	Khan et al. 2016
Serbia	2014	4	n/a	103	44.5	121.25	61.5	n/a	Kuzmanoski et al. 2014
USA, Washington	2017	14	0.07	25.8	12.5	54	n/a	19	Nezat et al. 2017
Australia	2018	1	0.13	12.4	82.3	125	5.45	253	Rate 2018
Brazil	2017	19	n/a	70.8	3.49	35.7	2.51	30.6	Gredilla et al. 2017
USA, Ohio	2018	1	0.17	54.21	14.92	75.72	29.83	20.38	Dietrich et al. 2018
Irish	2014	1	n/a	n/a	31.2	107.4	n/a	80.5	Dao et al. 2014
Poland	2017	1	0.8	16.3	55.5	176.7	10.5	120.2	Gąsiorek et al. 2017
Turkey	2016	16	1.053	54.67	37.54	91.92	39.23	38.98	Goksel et al. 2016
China, Wuhan*	2020	78	4.46	58.74	31.39	186.28	27.00	34.89	This study
China, Beijing	2019	121	0.49	63.57	35.49	145.7	27.12	27.56	Liu et al. 2020
China, Urumqi	2018	15	0.714	59.14	42.54	103.7	21.27	n/a	Zhang et al. 2018
China, Guangzhou	2017	18	0.2	53.1	63.7	91.5	25.4	110.6	Gu et al. 2017
China, Changchun	2015	12	0.325	59.28	37.82	169.26	23.08	69.12	Liu et al. 2015
China, Shanghai	2019	10	0.44	59.14	29.05	103.7	21.27	n/a	Wang et al. 2018

n/a, data not available.

Table S7 Summary statistics for non-carcinogenic and carcinogenic health risk range based on Monte Carlo simulation using

Risk	HMs	Mean（median）		SD		95%CI	
		Adult	Children	Adult	Children	Adult	Children
HQ	Cd	8.83E-3 (1.81E-3)	1.64E-2 (4.77E-3)	3.34E-2	4.59E-2	6.43E-5，6.20E-2	2.24E-4，1.10E-1
	Cr	2.43E-2（1.53E-2）	6.01E-2（5.45E-2）	3.29E-2	2.88E-2	4.49E-3，9.86E-2	2.42E-2，1.27E-1
	Cu	3.41E-4 (3.03E-4)	1.94E-3（1.79E-3）	1.89E-4	8.32E-4	9.41E-5，8.03E-4	7.89E-4，3.96E-3
	Zn	2.88E-4（2.54E-4）	1.55E-3 (1.44E-3)	1.69E-4	6.38E-4	8.22E-5，6.75E-4	6.58E-4，3.09E-3
	Ni	5.92E-4 (5.30E-4)	3.33E-3 (3.08E-3)	3.32E-4	1.41E-3	1.65E-4，1.38E-3	1.34E-3，6.76E-3
	Pb	4.87E-3 (4.16E-3)	2.50E-2（2.27E-2）	3.43E-3	1.14E-2	1.27E-3，1.23E-2	9.84E-3，5.29E-2
HI	Total	3.92E-2（2.48E-2）	1.08E-1（9.64E-2）	5.62E-2	6.10E-2	7.92E-3，1.61E-1	4.75E-2，2.37E-1
CR	Cr	5.82E-7 (5.43E-7)	1.50E-6（1.40E-6）	2.38E-7	5.85E-7	2.40E-7，1.17E-6	6.45E-7，2.91E-6
	Ni	3.61E-9（3.29E-9）	2.64E-9（2.42E-9）	1.65E-9	1.20E-9	1.40E-9，7.70E-9	1.00E-9，5.57E-9
	Pb	1.11E-7（9.98E-8）	7.20E-7（6.53E-7）	6.11E-8	3.28E-7	2.81E-8，2.64E-7	2.83E-7，1.52E-6
	Cd	1.02E-5（2.96E-6）	6.60E-5（1.99E-5）	2.94E-5	1.79E-4	1.29E-7，6.55E-5	9.96E-7，4.32E-4
TCR	Total	1.09E-5（3.66E-6）	6.82E-5（2.22E-5）	2.95E-5	1.79E-4	6.47E-7， 6.61E-5	2.92E-6，4.35E-4

Crystal Ball (vs. 11.1.2.4).

Abbreviations: HQ, hazard quotient of every heavy metal(loid)s; HI, Hazard index posed by multiple heavy metal(loid)s; CR, cancer risk of every heavy metal(loid)s; TCR, total cancer risks posed by multiple heavy metal(loid)s.

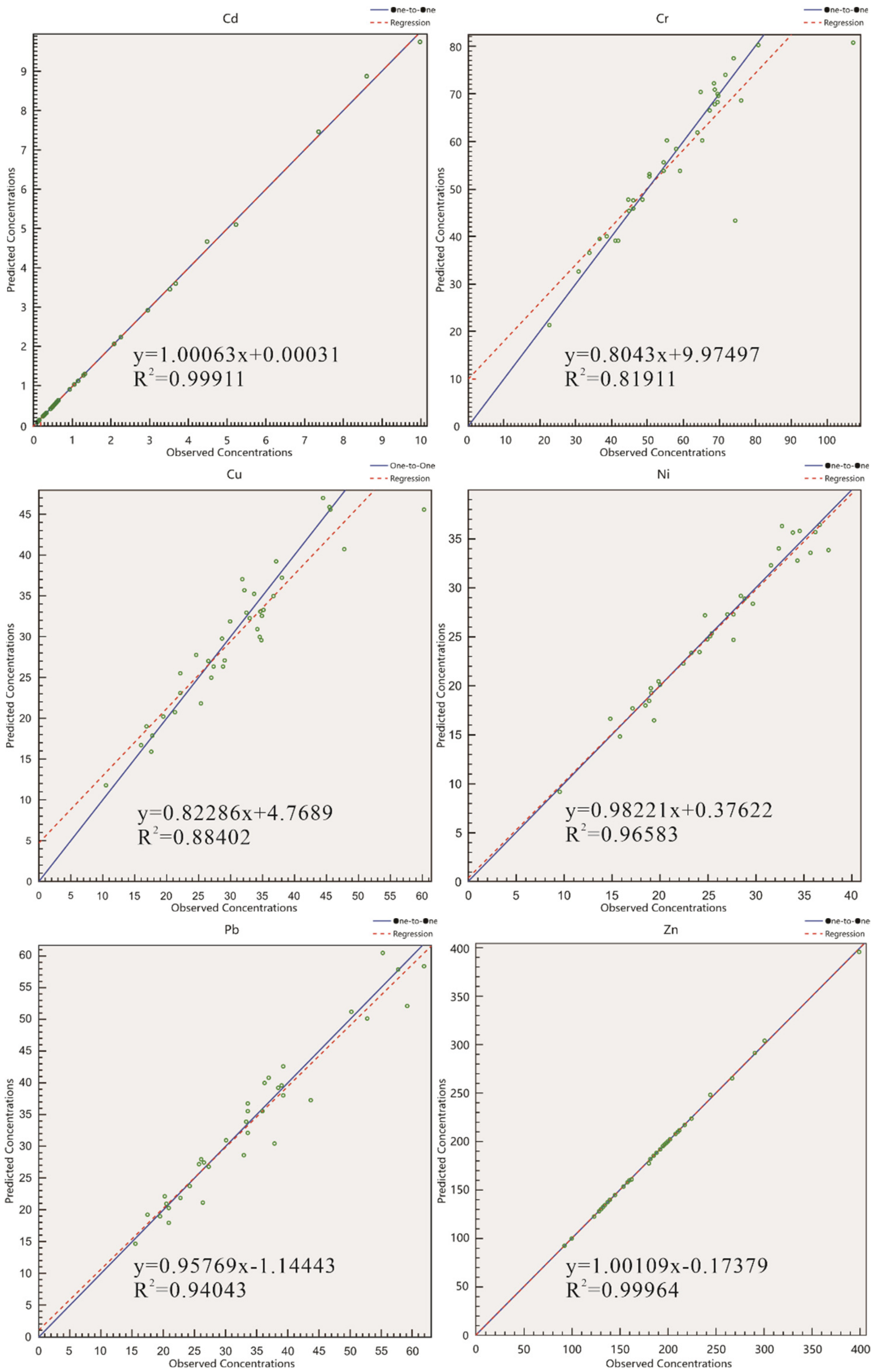

Figure S1. Fitting coefficients (R^2) between soil heavy metal(loid)s observed concentrations and predicted concentrations by PMF model.

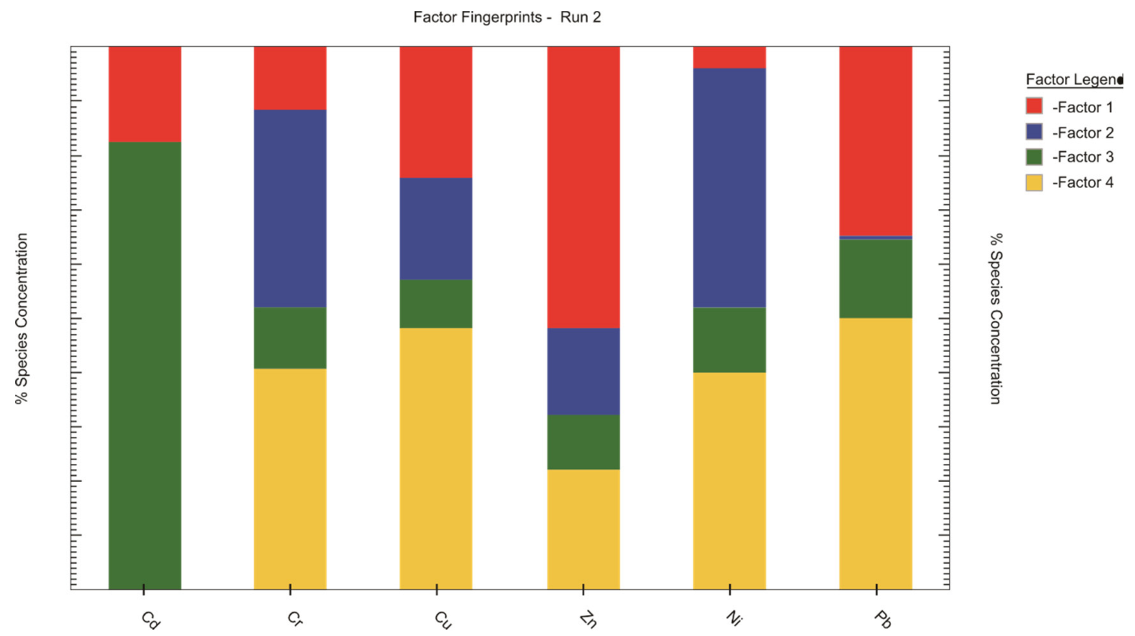

Figure S2. Factor contributions of soil heavy metal(loid)s from PMF model

References

1. Ferreira-Baptista, L. and De Miguel, E. (2005) Geochemistry and risk assessment of street dust in Luanda, Angola: A tropical urban environment. Atmospheric Environment Vol.39, 4501-4512.
2. Gu, Y.G., Lin, Q. and Gao, Y.P. (2017) Metals in exposed-lawn soils from 18 urban parks and its human health implications in southern China's largest city, Guangzhou. Journal of Cleaner Production Vol.163, 164-171.
3. USEPA, 2019. Environmental Protection Agency, Region 3, Risk Based Screening Tables (RSLs). Unites States Environmental Protection Agency, Washington, DC.
4. Yang, S., Zhao, J., Chang, S.X., Collins, C., Xu, J. and Liu, X. (2019) Status assessment and probabilistic health risk modeling of metals accumulation in agriculture soils across China: A synthesis. Environment International Vol.128, 165-174.
5. USDoE., 2011. The Risk Assessment Information System (RAIS). U.S. Department of Energy's Oak Ridge.
6. USEPA., 2011. Exposure Factors Handbook (Final Edition), U.S. Environment Protection Agency, Washington, DC.
7. Zhang, Y.Q., Wang, S.T., Gao, Z.J., et al., 2021. Contamination characteristics, source analysis and health risk assessment of heavy metals in the soil in Shi River Basin in China based on high density sampling. Ecotoxicology and Environmental Safety. 227, 112926.
8. MEP, 2013. Ministry of environmental protection of the People's republic of China (MEP). Exposure Factors Handbook of Chinese Population. China Environmental Science Press.
9. Liu, J., Wang, Y.N., Liu, X.M., et al., 2021. Occurrence and health risks of heavy metals in plastic-shed soils and vegetables across China. *Agriculture, Ecosystems & Environment*. 321, 107632.
10. Chen, H., Teng, Y., Lu, S., et al., 2016. Source apportionment and health risk assessment of trace metals in surface soils of Beijing metropolitan, China. *Chemosphere*, 144, 1002-1011.
11. Chen, H.R., Wang, L., Hu, B.F., et al., 2022. Potential driving forces and probabilistic health risks of heavy metal accumulation in the soils from an e-waste area, southeast China. *Chemosphere*. 289, 133182.
12. USEPA., 2011. Exposure Factors Handbook (Final Edition), U.S. Environment Protection Agency, Washington, DC.
13. Yang, Q.C., Zhang, L.M., Wang, H.L., et al., 2022. Bioavailability and health risk of toxic heavy metals (As, Hg, Pb and Cd) in urban soils: A Monte Carlo simulation approach. *Environmental Research.* 214, 1, 113772.
14. Kim, W., Song, I., Shin, J., Oh, C., Kim, E., Kim, K., Kim, H., Kim, J. and Choi, Y. (2017) A Study on Soil Contamination of Children`s Parks within the Gyeonggi-do Province Area. Korean Journal of Environmental Health Sciences Vol.43(No.3), 233-239.
15. Brtnicky, M., Pecina, V., Hladky, J., Radziemska, M., Koudelkova, Z., Klimanek, M., Richtera, L., Adamcova, D., Elbl, J., Galiova, M.V., Balakova, L., Kynicky, J., Smolikova, V., Houska, J., Vaverkova, M.D., 2019. Assessment of phytotoxicity, environmental and health risks of historical urban park soils. Chemosphere 220, 678-686.
16. Khan, S., Munir, S., Sajjad, M. and 1, G.L. (2016) Urban park soil contamination by potentially harmful elements and human health risk in Peshawar City, Khyber Pakhtunkhwa, Pakistan. Journal of Geochemical Exploration Vol.165, 102-110.

17. Kuzmanoski, M.M., Todorović, M.N., Aničić Urošević, M.P. and Rajšić, S.F. (2014) Heavy metal content of soil in urban parks of Belgrade. Hemijska Industrija Vol.68(No.5), 643-651.
18. Nezat, C.A., Hatch, S.A. and Uecker, T. (2017) Heavy metal content in urban residential and park soils: A case study in Spokane, Washington, USA. Applied Geochemistry Vol.78, 186-193.
19. Rate, A.W. (2018) Multielement geochemistry identifies the spatial pattern of soil and sediment contamination in an urban parkland, Western Australia. Science of the Total Environment 627, 1106-1120.
20. Gredilla, A., Fdez-Ortiz de Vallejuelo, S., Gomez-Nubla, L., Carrero, J., Le, atilde, o, F., Madariaga, J. and Silva, L. (2017) Are children playgrounds safe play areas? Inorganic analysis and lead isotope ratios for contamination assessment in recreational (Brazilian) parks. Environmental Science & Pollution Research Vol.24(No.31), 24333-24345.
21. Dietrich, M., Huling, J. and Krekeler, M.P.S. (2018) Metal pollution investigation of Goldman Park, Middletown Ohio: Evidence for steel and coal pollution in a high child use setting. Science of the Total Environment Vol.618, 1350-1362.
22. Dao, L., Morrison, L., Zhang, H. and Zhang, C. (2014) Influences of traffic on Pb, Cu and Zn concentrations in roadside soils of an urban park in Dublin, Ireland. Environmental Geochemistry & Health Vol.36(No.3), 333-343.
23. Gąsiorek, M., Kowalska, J., Mazurek, R. and Pająk, M. (2017) Comprehensive assessment of heavy metal pollution in topsoil of historical urban park on an example of the Planty Park in Krakow (Poland). Chemosphere Vol.179, 148-158.
24. Goksel, D., Kurtulus, O.H., Huseyin, O., Osman, P.A., Ilker, O. and Muhammet, B. (2016) Heavy metal concentrations of selected public parks of Istanbul City. MATEC Web of Conferences Vol.64, 2001.
25. Liu, L.L., Liu, Q., Ma, J. and Wu, H. (2020) Heavy metal(loid)s in the topsoil of urban parks in Beijing, China: Concentrations,potential sources, and risk assessment. Environmental Pollution Volume 260, 114083.
26. Zhang, Z.Y., Yang, X.D., Simay, Z. and Mohammed, A. (2018) Health risk evaluation of heavy metals in green land soils from urban parks in Urumqi, northwest China. Environmental Science and Pollution Research 25(5), 4459-4473.
27. Gu, Y.-G., Lin, Q. and Gao, Y.-P. (2017) Reprint of: Metals in exposed-lawn soils from 18 urban parks and its human health implications in southern China's largest city, Guangzhou. Journal of Cleaner Production Vol.163, S164-S171.
28. Liu, Q., Wang, Y., Liu, J., Wang, Q. and Zou, M. (2015) Grain-size distribution and heavy metal contamination of road dusts in urban parks and squares in Changchun, China. Environmental Geochemistry & Health 37(1), 71-82.
29. Wang, L.N., Chang, J., Zheng, X.R., et al., 2018. Survey of ecological environmental conditions and influential factors for public parks in Shanghai. Chemosphere. 190, 9-16.
